# Sleep Difficulties and Their Associations With Smoking Abstinence Among Adults Seeking Tobacco Cessation Treatment

**DOI:** 10.1016/j.amepre.2026.108342

**Published:** 2026-03-18

**Authors:** Chaelin K. Ra, Andrea C. Villanti, Michelle T. Bover-Manderski, Melissa Mercincavage, Michael B. Steinberg

**Affiliations:** 1 Rutgers Cancer Institute, Robert Wood Johnson Medical School, Rutgers University, New Brunswick, New Jersey; 2 Rutgers Institute for Nicotine & Tobacco Studies, Rutgers University, New Brunswick, New Jersey; 3 Department of Health Behavior, Society and Policy, Rutgers School of Public Health, Piscataway, New Jersey; 4 Department of Biostatistics & Epidemiology, Rutgers School of Public Health, Piscataway, New Jersey

## Abstract

**Introduction:**

In prior studies, sleep difficulties have been associated with lower likelihood of smoking cessation, although little is known about this relationship among people trying to quit smoking. This study aimed to examine the associations between pre-existing sleep difficulties and smoking cessation outcomes in adults seeking tobacco cessation treatment.

**Methods:**

Participants in a New Jersey statewide cessation program between November 2019 and July 2022 provided self-reported smoking abstinence at 1-month (n=2,919) and/or 7-month (n=1,471) follow-ups. Participants completed 3 items on sleep difficulties at baseline: difficulty sleeping (past and present) and insomnia as a withdrawal symptom. Multivariable logistic regression models estimated the associations between sleep difficulties and smoking cessation at each follow-up, controlling for sex, age, household income, ethnicity, and baseline heaviness of smoking index in 2024.

**Results:**

At baseline, 3.6% of participants reported difficulty sleeping in the past, 20.1% reported difficulty sleeping in the present, and 14.6% experienced insomnia as a withdrawal symptom. Lower income, depression, anxiety, excessive alcohol and caffeine use, dual use, and identifying as male or Hispanic were associated with higher odds of pre-existing sleep difficulties (e.g., OR=4.30, 95% CI=3.66, 5.04). Sleep difficulty in the past was positively associated with nonabstinence at 1-month follow-up (AOR=2.95, 95% CI=1.28, 6.79) and 7 months (AOR=4.64, 95% CI=1.06, 20.23). There were no associations between the other 2 measures of sleep difficulties and cessation outcomes.

**Conclusions:**

Pre-existing sleep difficulties may be associated with lower short-term smoking abstinence among adults seeking tobacco cessation treatment. Addressing pre-existing sleep difficulties in smoking cessation interventions may enhance the chances of successful smoking cessation.

## INTRODUCTION

Tobacco use remains the leading preventable cause of morbidity and mortality in the U.S.,^1,2^ and although most people who smoke want to quit, fewer than 5% make a successful quit attempt.^3^ Sleep difficulties, including insomnia, insufficient sleep duration, and poor sleep quality, have been associated with smoking more cigarettes per day,^4^ greater nicotine dependence,^5^ higher withdrawal,^6^ and more smoking relapses.^7,8^ Meanwhile, sleep problems are also more common among adults with lower levels of education or income,^9–16^ depression, anxiety,^17,18^ or excessive caffeine^19^ and alcohol use.^20^ Smoking cessation remains a critical public health concern, and sleep difficulties may play an important role in smoking cessation, yet this association is not well explored.

This study aimed to investigate sociodemographic, behavioral, and psychological factors related to pre-existing sleep difficulties and to examine the impact of sleep difficulties on smoking abstinence at 1-month and 7-month follow-ups among adults seeking tobacco cessation treatment.

## METHODS

Participants were adults (aged ≥18 years) seeking smoking cessation treatment at 11 Quitcenters in New Jersey between November 2019 and July 2022. During their baseline visit (in person or online), trained clinicians conducted standardized interviews to collect information on sociodemographic characteristics, health behaviors, medical history, and tobacco-related questions. Deidentified assessment data were entered into a secure, cloud-based database (Datstat) maintained by the New Jersey Department of Health. Participants provided self-reported smoking cessation outcomes at 1-month or 7-month follow-ups to assess short- and long-term abstinence status. The final sample included participants who returned at either 1-month or 7-month follow-ups. Those with missing smoking outcome data either at follow-up or with incomplete baseline sleep records were excluded from the primary analytic sample; however, these cases were examined separately, and the results are presented in the Appendices (available online). This study was approved by the Rutgers IRB (Pro2020002102). All participants were provided written informed consent prior to participation in the study.

All participants answered 3 items on sleep difficulties at baseline: having difficulty sleeping in the past, having difficulty sleeping in the present, and experiencing insomnia as a withdrawal symptom in the past. Each response was dichotomized into 0 (no) and 1 (yes). The predictors were the 3 respective pre-existing sleep difficulties, sleep difficulties in the past and/or present, and endorsing any sleep problems across the 3 variables.

The Heaviness of Smoking Index^21^ and awake at night to smoke (measures of smoking dependence) were assessed at baseline, as was current use of tobacco and nicotine products (e.g., cigars, E-cigarettes). Current use was categorized as cigarette only, vaping only (E-cigarette), or dual use of both. Smoking status at follow-up was self-reported (abstinence in the previous 7 days versus smoked in the past 7 days).

Sociodemographic characteristics assessed included age, sex, race, Hispanic ethnicity, education, income, and marital status. Under the medical history section, participants self-reported depression, anxiety, excessive alcohol use, and excessive caffeine (e.g., caffeine in excess) use, which were self-reported for both the past and present. Responses for the past and present were combined and dichotomized for each variable.

Descriptive statistics were used to characterize the sample, and t-tests and Fisher’s exact chi-square tests were conducted to test bivariate associations (Tables 1 and 2 and Appendix Table 1 and 2, available online). Multivariable logistic regression models estimated the associations between pre-existing sleep difficulties and smoking cessation at 1-month and 7-month follow-ups, controlling for sex, age, ethnicity, level of education, baseline heaviness of smoking index, depression, anxiety, excessive alcohol use, and excessive caffeine use (Table 3 and Appendix Table 3, available online [sensitivity analyses using 2 additional datasets: first, no missing responses for the pre-existing sleep difficulties variables and, second, no missing responses for continued tobacco use]). All analyses were performed using SPSS (29.0.2.0). This analysis was designed to be exploratory and not intended to establish causality.

## RESULTS

Participants had an average age of 50.9 (SD=13.7) years; 52.3% were male, 71.7% were non-Hispanic, and 61.3% were White (Table 1). At baseline, higher income, identifying as Female and non-Hispanic, and being never married were associated with lower odds of pre-existing sleep difficulties (ORs=0.22~0.72, 95% CI=0.16, 0.85), and depression, anxiety, excessive alcohol and caffeine use, awake at night, dual use, and higher Heaviness of Smoking Index were associated with higher odds of pre-existing sleep difficulties (ORs=1.19~4.30, 95% CI=1.13, 5.04) (Appendix Table 1, available online). In addition, there were significant differences in sociodemographic characteristics (i.e., level of education, income, ethnicity, marital status, and age) between the analytic sample (n=3,295) and lost to follow-up (n=2,657) data (Appendix Table 2, available online).

In Table 2, when compared with smoking difficulties and abstinence status (any tobacco versus abstinence), only having sleep difficulties in the past was significantly related to the abstinence status at the 7-month follow-up (p=0.006). In multivariable models, sleep difficulty in the past was positively associated with nonabstinence at 1-month follow-up (AOR=2.95, 95% CI=1.28, 6.79) and 7 months (AOR=4.64, 95% CI=1.06, 20.23) (Table 3). There were no associations between the other 2 measures of sleep difficulties and cessation outcomes. Results were consistent in sensitivity analyses with missing data (Appendix Table 3, available online).

## DISCUSSION

This study used a large sample of treatment-seeking adults who smoke cigarettes to better understand the relationship between sleep problems and smoking abstinence during the process of quitting. Depression, anxiety, and identifying as non-Hispanic were related to higher rates of sleep difficulties in the past and present and insomnia as a withdrawal symptom. In addition, past sleep difficulties but not present sleep difficulties or insomnia due to nicotine withdrawal were associated with lower odds of abstinence at both the 1-month and 7-month follow-ups.

Findings support previous studies indicating that sleep difficulties, including insomnia, may hinder smoking abstinence.^22,23^ Studies have shown that individuals with sleep problems are more likely to relapse because these disturbances can worsen withdrawal symptoms.^22,23^ Recent work found that greater nicotine dependence was associated with poorer sleep quality among treatment-seeking adults who smoke, further reinforcing the interconnection between tobacco use and sleep problems.^24^ In this study, past sleep difficulties were significantly associated with abstinence status at the 1-month and 7-month follow-ups, suggesting that pre-existing sleep issues may increase the risk of relapse and make it harder to maintain smoking cessation.^8^

However, prior research did not distinguish pre-existing sleep conditions. In this study, the associations between current sleep difficulties, insomnia as a withdrawal symptom, and abstinence at the 1-month and 7-month follow-ups were not significant. These discrepancies from prior findings may reflect measurement limitations—such as unclear distinctions between past and present sleep conditions and the lack of data on sleep duration or quality, which are known to influence cessation outcomes.

### Limitations

Nicotine’s effects on sleep-regulating neurotransmission and the transient sleep disturbances associated with some cessation medications may also influence the observed associations.^25,26^ Other limitations include the use of self-reported abstinence, the small proportion (3.6%) of participants reporting past sleep difficulties, and recruitment from a single state, which suggest that these findings should be interpreted with caution.^27^ Future studies should incorporate detailed sleep measures (e.g., sensors) and assess other sleep disturbances, such as parasomnias. Although this analysis focused on pre-existing sleep difficulties, the relationship between smoking and poor sleep is likely bidirectional—sleep disturbances may both result from and contribute to ongoing tobacco use and relapse.

## CONCLUSIONS

Cigarette smoking and sleep problems are prevalent in the general population and independently impact health. Pre-existing sleep difficulties may be related to lower smoking abstinence among adults seeking tobacco cessation treatment. The role of sleep difficulties in smoking cessation interventions has been understudied but may be important for improving smoking cessation outcomes. Addressing pre-existing sleep difficulties in smoking cessation treatment could help reduce relapse risk and enhance cessation success. Future studies should incorporate more detailed and objective sleep assessments to clarify the directionality and underlying mechanisms of the association between smoking and sleep difficulties.

## Supplementary Material

Supplementary Material

Supplemental materials associated with this article can be found in the online version at https://doi.org/10.1016/j.amepre.2026.108342 .

## Figures and Tables

**Table 1.** Relations Between Sociodemographic Characteristics, Medical History, and Tobacco Use and Sleep-Related Variables (*n*=3,295)

Characteristics	Difficulty sleeping, past			Difficulty sleeping, present			Insomnia as a withdrawal symptom		
	Yes *n*=120^a^	%	*p*-value	Yes *n*=663^a^	%	*p*-value	Yes *n*=481 ^a^	%	*p*-value
Overall %	3.64%			20.12%			14.60%		
Education									
Less than high school	7	3.3%	0.013	69	23.7%	0.001	55	18.7%	0.150
High school or GED	30	3.5%		205	17.8%		153	13.5%	
Some college	53	5.0%		258	24.1%		160	15.0%	
College or higher	27	4.4%		115	18.7%		94	15.7%	
Income									
<15,000	24	3.5%	0.231	217	31.5%	<0.001	155	22.9%	<0.001
15,000–30,000	35	3.8%		137	14.9%		104	11.4%	
30,000–45,000	8	1.8%		40	9.1%		28	6.3%	
45,000–80,000	6	2.6%		42	18.1%		34	14.4%	
>80,000	11	4.7%		65	28.0%		41	18.8%	
Sex									
Male	53	3.9%	0.055	267	19.8%	0.001	200	15.0%	0.004
Female	32	2.6%		181	14.6%		136	11.1%	
Ethnicity									
Hispanic	16	2%	0.001	99	11.9%	<0.001	100	12.1%	0.006
Non-Hispanic	102	4%		551	23.9%		364	16.1%	
Race									
White	82	4.1%	0.422	475	24.0%	<0.001	315	16.1%	<0.001
Black	18	3.1%		107	18.5%		101	18.0%	
Other	14	3.2%		44	10.2%		36	8.4%	
Marital status									
Currently married	62	4.5%	0.115	271	19.9%	<0.001	249	18.4%	<0.001
Never married	37	3.2%		203	17.8%		141	12.6%	
Other (divorced, widowed)	20	3.0%		178	26.5%		79	12.0%	
Depression									
No	28	1.4%	<0.001	236	11.4%	<0.001	219	11.1%	<0.001
Yes	92	7.9%		424	36.6%		258	22.7%	
Anxiety									
No	25	1.3%	<0.001	237	12.2%	<0.001	201	10.8%	<0.001
Yes	95	7.4%		424	33.1%		276	22.0%	
Excessive alcohol use									
No	39	4.5%	<0.001	240	27.6%	0.01	161	20.8%	0.335
Yes	42	9.7%		150	34.6%		95	23.2%	
Excessive caffeine use									
No	92	6.1%	0.577	418	27.5%	<0.001	289	20.6%	0.008
Yes	25	5.2%		201	41.8%		124	26.4%	
Awake at night to smoke									
No	79	3.8%	1.000	345	16.8%	<0.001	215	10.6%	<0.001
Yes	40	3.8%		302	28.4%		248	23.5%	
Dual use									
Cigarette only	86	3.2%	<0.001	529	19.5%	<0.001	395	14.7%	0.092
Vaping only	5	4.1%		23	19.0%		18	15.4%	
Both	21	8.5%		86	35.0%		49	19.9%	
	Yes (mean/SD)	No (mean/SD)	*p*-value	Yes (mean/SD)	No (mean/SD)	*p*-value	Yes (mean/SD)	No (mean/SD)	*p*-value
Age, year	47.22 (15.16)	50.94 (13.65)	0.004	50.44 (13.04)	50.90 (13.89)	0.442	48.62 (13.84)	51.23 (13.73)	<0.001
HSI	2.73 (1.53)	2.65 (1.57)	0.609	3.09 (1.53)	2.54 (1.56)	<0.001	2.60 (1.55)	2.99 (1.61)	<0.001

*Note*: *p*-values were from *t*-tests, and Fisher’s exact chi-square tests were conducted to test bivariate associations.

a Numbers for each variable do not add to the total owing to missing responses.

HIS, Heaviness of Smoking Index.

**Table 2.** Frequencies and Weighted Distributions of the 3 Pre-Existing Sleep Difficulties, Stratified by Abstinence Status at 1-Month and 7-Month Follow-Ups

Variables	At 1-month follow-up							At 7-month follow-up						
	*n*	Abstinence *n* (%)	*p*-value^a,b^	Continued tobacco use			*p*-value^a,c^	*n*	Abstinence n (%)	*p*-value^a,b^	Continued tobacco use			*p*-value^a,c^
				Any tobacco	E-cigarette	Both					Any tobacco	E-cigarette	Both	
Difficulty sleeping, past														
No	2,770	797 (28.8%)	0.086	1,820 (65.7%)	81 (2.9%)	72 (2.6%)	0.053	1,361	335 (24.6%)	0.006	905 (66.5%)	48 (3.5%)	73 (5.4%)	0.001
Yes	97	20 (20.6%)		67 (69.1%)	<5 ^d^	6 (6.2%)		50	<5 ^d^		35 (70%)	<5 ^d^	8 (16%)	
Difficulty sleeping, present														
No	2,304	651 (28.3%)	0.567	1,531 (66.4%)	71 (3.1%)	51 (2.2%)	0.01	1,102	269 (24.4%)	0.547	741 (67.2%)	37 (3.4%)	55 (5.0%)	0.096
Yes	563	166 (29.5%)		356 (63.2%)	14 (2.5%)	27 (4.8%)		309	70 (22.7%)		199 (64.4%)	14 (4.5%)	26 (8.4%)	
Difficulty sleeping (past or present)														
No	2,215	632 (28.5%)	0.961	1,469 (66.3%)	68 (3.1%)	46 (2.1%)	0.003	1,056	267 (25.3%)	0.062	707 (67.0%)	34 (3.2%)	48 (4.5%)	0.002
Yes	652	185 (28.4%)		418 (64.1%)	16 (2.6%)	32 (4.9%)		355	72 (20.3%)		233 (65.6%)	17 (4.8%)	33 (9.3%)	
Insomnia as a withdrawal symptom														
No	2,400	667 (27.8%)	0.061	1,598 (66.6%)	75 (3.1%)	60 (2.5%)	0.036	1,186	283 (23.9%)	0.29	801 (67.5%)	39 (3.3%)	63 (5.3%)	0.106
Yes	421	136 (32.3%)		262 (62.2%)	7 (1.7%)	16 (3.8%)		204	56 (27.5%)		122 (59.8%)	10 (4.9%)	16 (7.8%)	
Any sleep issue (difficulty past or present, insomnia as a withdrawal symptom)														
No	1,912	534 (27.9%)	0.554	1,282 (67.1%)	60 (3.1%)	36 (1.9%)	0.001	890	215 (24.2%)	0.588	614 (69.0%)	26 (2.9%)	35 (3.9%)	<0.001
Yes	864	251 (29.1%)		553 (64.0%)	21 (2.4%)	39 (4.5%)		452	103 (22.8%)		284 (62.8%)	21 (4.6%)	44 (9.7%)	

a Fisher’s exact test was performed.

b Abstinence versus continued tobacco use (nonabstinence).

c Abstinence versus any tobacco versus E-cigarette versus both.

d Cell counts <5 and corresponding percentages are suppressed to protect participant privacy.

**Table 3.** Results From Multivariable Models Examining the Associations Between Pre-Existing Sleep Difficulties and Continued Tobacco Use at 1- and 7-Month Follow-Ups

Variables	Continued tobacco use at 1-month follow-up			Continued tobacco use at 7-month follow-up		
	*n* (%)	Unadjusted OR (95% CI)	Adjusted OR (95% CI)	*n* (%)	Unadjusted OR (95% CI)	Adjusted OR (95% CI)
Difficulty sleeping, past						
No	1,973 (71.2%)	ref	ref	No 1026 (75.4%)	ref	ref
Yes	77 (79.4%)	1.56 (0.94, 2.56)	**2.95 (1.28, 6.79)**	Yes 46 (92.0%)	**3.76 (1.34, 10.51)**	**4.64 (1.06, 20.23)**
Difficulty sleeping, present						
No	1,653 (71.7%)	ref	ref	No 833 (75.6%)	ref	ref
Yes	397 (70.5%)	0.94 (0.77, 1.15)	0.88 (0.62, 1.24)	Yes 239 (77.3%)	1.10 (0.82, 1.49)	0.85 (0.54, 1.34)
Difficulty sleeping (past or present)						
No	1,583 (71.5%)	ref	ref	No 789 (74.7%)	ref	ref
Yes	467 (71.6%)	1.01 (0.83, 1.23)	1.11 (0.80, 1.55)	Yes 283 (79.7%)	1.33 (0.99, 1.79)	1.09 (0.70, 1.71)
Insomnia as a withdrawal symptom						
No	1,733 (72.2%)	ref	ref	No 903 (76.1%)	ref	ref
Yes	285 (67.7%)	0.81 (0.65, 1.01)	0.83 (0.56, 1.21)	Yes 148 (72.5%)	0.83 (0.59, 1.16)	0.85 (0.50, 1.45)
Any sleep issue (difficulty past or present, insomnia as a withdrawal symptom)						
No	1,378 (72.1%)	ref	ref	No 675 (75.8%)	ref	ref
Yes	613 (70.9%)	0.95 (0.79, 1.13)	1.10 (0.80, 1.52)	Yes 349 (77.2%)	1.08 (0.83, 1.41)	0.92 (0.59, 1.43)

*Note*: Boldfaces indicate statistical significance (*p*<0.05).

Models were adjusted for sex, age, education, ethnicity, baseline heaviness of smoking index, depression, anxiety, excessive alcohol use, and excessive caffeine use. Continued smoking refers to nonabstinence at each follow-up.

## References

[1] Prochaska JJ, Das S, Young-Wolff KC. Smoking, mental illness, and public health. Annu Rev Public Health. 2017;38:165–185. 10.1146/annurev-publhealth-031816-044618. 27992725 PMC5788573

[2] Gfroerer J, Dube SR, King BA, Garrett BE, Babb S and McAfee T, Vital Signs: current cigarette smoking among adults aged≥ 18 years with mental illness—United States, 2009-2011, MMWR Morb Mortal Wkly Rep, 62 (5), 2013, 81. https://pmc.ncbi.nlm.nih.gov/articles/PMC4604817/. Accessed March 6, 2025. 23388551 PMC4604817

[3] Wang TW, Asman K, Gentzke AS, Tobacco product use among adults—United States, 2017. MMWR Morb Mortal Wkly Rep. 2018;67 (44):1225–1232. 10.15585/mmwr.mm6744a2. 30408019 PMC6223953

[4] Warren CM, Riggs NR, Pentz MA Longitudinal relationships of sleep and inhibitory control deficits to early adolescent cigarette and alcohol use. J Adolesc. 2017;57:31–41. 10.1016/j.adolescen-ce.2017.03.003. 28334632 PMC5436806

[5] Cohen A, Ben Abu NB, Haimov I The interplay between tobacco dependence and sleep quality among young adults. Behav Sleep Med. 2018;18(2):163–176. 10.1080/15402002.2018.1546707. 30463440

[6] Purani H, Friedrichsen S, Allen AM Sleep quality in cigarette smokers: associations with smoking-related outcomes and exercise. Addict Behav. 2019;90:71–76. 10.1016/j.addbeh.2018.10.023. 30368021 PMC6324958

[7] Patterson F, Grandner MA, Malone SK, Rizzo A, Davey A, Edwards DG Sleep as a target for optimized response to smoking cessation treatment. Nicotine Tob Res. 2019;21(2):139–148. 10.1093/ntr/ntx236. 29069464 PMC6329404

[8] Matthews JA, Sallis HM, Dyer ML, McConville R, Isotalus H, Attwood AS Associations between self-reported sleep quality, fatigue severity, factors associated with successful cessation, and cessation beliefs among regular smokers. Nicotine Tob Res. 2024;26(7):835–842. 10.1093/ntr/ntad231. 37996095 PMC11190051

[9] Grandner MA, Patel NP, Gehrman PR, Who gets the best sleep? Ethnic and socioeconomic factors related to sleep complaints. Sleep Med. 2010;11(5):470–478. 10.1016/j.sleep.2009.10.006. 20388566 PMC2861987

[10] Xiao Q, Hale L Neighborhood socioeconomic status, sleep duration, and napping in middle-to-old aged U.S. men and women. Sleep. 2018;41(7):zsy076. 10.1093/sleep/zsy076. 29697844 PMC6047422

[11] Hale L, Do DP Racial differences in self-reports of sleep duration in a population-based study. Sleep. 2007;30(9):1096–1103. 10.1093/sleep/30.9.1096. 17910381 PMC1978399

[12] Hale L Who has time to sleep? J Public Health (Oxf). 2005;27(2):205–211. 10.1093/pubmed/fdi004. 15749721

[13] Lauderdale DS, Knutson KL, Yan LL, Objectively measured sleep characteristics among early-middle-aged adults: the CARDIA study. Am J Epidemiol. 2006;164(1):5–16. 10.1093/aje/kwj199. 16740591

[14] Patel SR Social and demographic factors related to sleep duration. Sleep. 2007;30(9):1077–1078. 10.1093/sleep/30.9.1077. 17910376 PMC1978396

[15] Patel SR, Malhotra A, Gottlieb DJ, White DP, Hu FB Correlates of long sleep duration. Sleep. 2006;29(7):881–889. 10.1093/sleep/29.7.881. 16895254 PMC3500381

[16] Adams J Socioeconomic position and sleep quantity in UK adults. J Epidemiol Community Health. 2006;60(3):267–269. 10.1136/jech.2005.039552. 16476759 PMC2465560

[17] Aernout E, Benradia I, Hazo JB, International study of the prevalence and factors associated with insomnia in the general population. Sleep Med. 2021;82:186–192. 10.1016/j.sleep.2021.03.028. 33957414

[18] Smagula SF, Stone KL, Fabio A, Cauley JA Risk factors for sleep disturbances in older adults: evidence from prospective studies. Sleep Med Rev. 2016;25:21–30. 10.1016/j.smrv.2015.01.003. 26140867 PMC4506260

[19] Gardiner C, Weakley J, Burke LM, The effect of caffeine on sub-sequent sleep: a systematic review and meta-analysis. Sleep Med Rev. 2023;69:101764. 10.1016/j.smrv.2023.101764. 36870101

[20] Zheng D, Yuan X, Ma C, Alcohol consumption and sleep quality: a community-based study. Public Health Nutr. 2021;24(15):4851–4858. 10.1017/S1368980020004553. 33183388 PMC11077453

[21] Heatherton TF, Kozlowski LT, Frecker RC, Rickert W, Robinson J Measuring the heaviness of smoking: using self-reported time to the first cigarette of the day and number of cigarettes smoked per day. Br J Addict. 1989;84(7):791–799. 10.1111/j.1360-0443.1989.tb03059.x. 2758152

[22] Short NA, Mathes BM, Gibby B, Oglesby ME, Zvolensky MJ, Schmidt NB Insomnia symptoms as a risk factor for cessation failure following smoking treatment. Addict Res Theory. 2017;25(1):17–23. 10.1080/16066359.2016.1190342. 29104521 PMC5665381

[23] Hagg SA, Ljunggren M, Janson C, Smokers with insomnia symptoms are less likely to stop smoking. Respir Med. 2020;170:106069. 10.1016/j.rmed.2020.106069. 32843184

[24] Ozden Sertcelik UO, Karalezli A The association between nicotine dependence and sleep quality in patients referred to a smoking cessation outpatient clinic: a cross-sectional study. Tob Induced Dis. 2024;22:1–9 (). 10.18332/tid/194170. PMC11533092 39498211

[25] Guzmân-Marín R, Alam MN, Mihailescu S, Szymusiak R, McGinty D, Drucker-Colín R Subcutaneous administration of nicotine changes dorsal raphe serotonergic neurons discharge rate during REM sleep. Brain Res. 2001;888(2):321–325. 10.1016/S0006-8993(00)03104-8. 11150492

[26] Ashare RL, Lerman C, Tyndale RF, Sleep disturbance during smoking cessation: withdrawal or side effect of treatment? J Smok Cessat. 2017;12(2):63–70. 10.1017/jsc.2016.11. 28553407 PMC5443250

[27] Scheuermann TS, Richter KP, Rigotti NA, Accuracy of self-reported smoking abstinence in clinical trials of hospital-initiated smoking interventions. Addiction. 2017;112(12):2227–2236. 10.1111/add.13913. 28834608 PMC5673569

