# Supplementary material for: Sleep Difficulties and Their Associations With Smoking Abstinence Among Adults Seeking Tobacco Cessation Treatment
Source: Am J Prev Med. Author manuscript; Available in PMC 2026 Jul 13. (PMC13356954; doi:10.1016/j.amepre.2026.108342)
Supplement: Supplementary Material [file NIHMS2188182-supplement-Supplementary_Material.docx]

**Appendices**

- Appendix Figure 1
- Appendix Table 1
- Appendix Table 2
- Appendix Table 3-1
- Appendix Table 3-2

**Appendix Figure 1. Venn diagram of three pre-existing sleep difficulty variables**


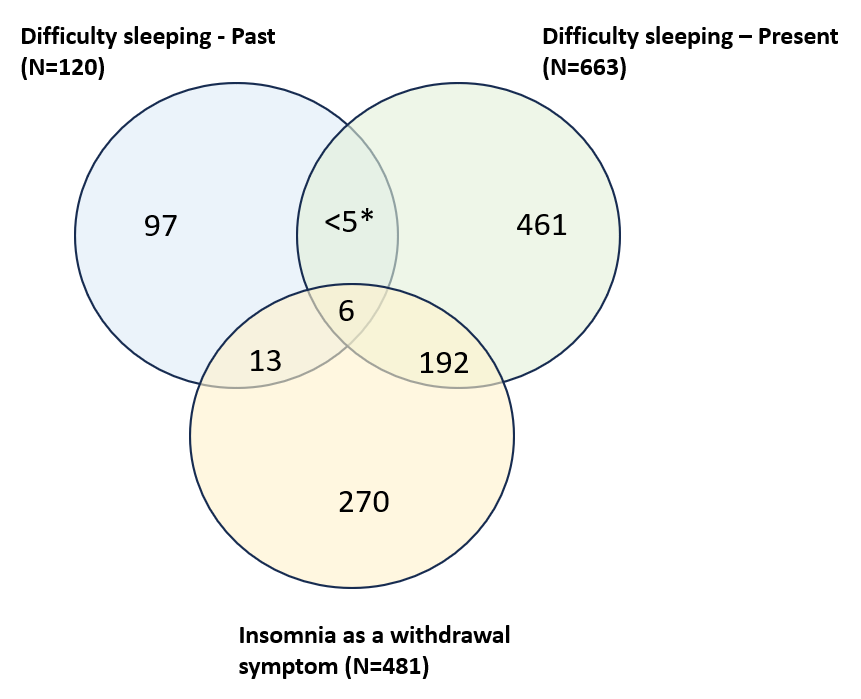


* Cell counts < 5 and corresponding percentages are suppressed to protect participant privacy.

**Appendix Table 1. Unadjusted Odds Ratios between sociodemographic characteristics, medical history, and tobacco use and sleep related variables**

|  | **Any sleep issue (difficulty past or present, insomnia as withdrawal symptom)** |
| --- | --- |
|  | **Unadjusted OR (95% CI)** |
| **Education** |  |
| Less than High School | ref. |
| High School or GED | 0.71 (0.54, 0.94) |
| Some college | 1.07 (0.81, 1.41) |
| College or higher | 0.97 (0.72, 1.31) |
| **Income** |  |
| <15,000 | ref. |
| 15,000-30,000 | 0.39 (0.31, 0.48) |
| 30,000-45,000 | 0.22 (0.16, 0.29) |
| 45,000-80,000 | 0.45 (0.31, 0.61) |
| >80,000 | 0.94 (0.69, 1.28) |
| **Gender** |  |
| Male | ref. |
| Female | 0.63 (0.53, 0.75) |
| **Ethnicity** |  |
| Hispanic | ref. |
| Non-Hispanic | 0.46 (0.38, 0.55) |
| **Race** |  |
| White | ref. |
| Black | 0.89 (0.73, 1.09) |
| Other | 0.37 (0.28, 0.48) |
| **Marital Status** |  |
| Currently married | ref. |
| Never married | 0.72 (0.60, 0.85) |
| Other (divorced, widowed) | 0.98 (0.80, 1.19) |
| **Depression** |  |
| No | ref. |
| Yes | 4.30 (3.66, 5.04) |
| **Anxiety** |  |
| No | ref. |
| Yes | 3.80 (3.25, 4.45) |
| **Excessive Alcohol Use** |  |
| No | ref. |
| Yes | 1.47 (1.15, 1.87) |
| **Excessive Caffeine Use** |  |
| No | ref. |
| Yes | 1.55 (1.25, 1.91) |
| **Awake at night to smoke** |  |
| No | ref. |
| Yes | 2.20 (1.88, 2.58) |
| **Dual Use** |  |
| Cig only | ref. |
| Vaping only | 1.01 (0.67, 1.51) |
| Both | 2.07 (1.59, 2.70) |
| **Age** | 0.99 (0.99, 1.00) |
| **HSI** | 1.19 (1.13, 1.25) |

| **Appendix Table 2. Sociodemographic characteristics, medical history, and tobacco use and sleep-related variables of analytic sample and lost to follow-up (*Total N=5952*)** | | | | | |
| --- | --- | --- | --- | --- | --- |
|  | **Analytic sample (N=3295)** | | **Lost to follow-up (N=2657)** | | ***P-value*** |
|  | **N** | **%** | **N** | **%** |  |
| **Education (N=5708)** |  |  |  |  |  |
| Less than High School | 296 | 9.3% | 300 | 11.9% | <.001 |
| High School or GED | 1170 | 36.6% | 1071 | 42.6% |  |
| Some college | 1096 | 34.3% | 791 | 31.5% |  |
| College or higher | 633 | 19.8% | 351 | 14.0% |  |
| **Income (N=4592)** |  |  |  |  |  |
| <15,000 | 701 | 27.5% | 647 | 31.6% | 0.01 |
| 15,000-30,000 | 934 | 36.7% | 708 | 34.6% |  |
| 30,000-45,000 | 444 | 17.4% | 320 | 15.6% |  |
| 45,000-80,000 | 236 | 9.3% | 209 | 10.2% |  |
| >80,000 | 232 | 9.1% | 161 | 7.9% |  |
| **Gender (N=4243)** |  |  |  |  |  |
| Male | 1386 | 52.3% | 856 | 53.7% | 0.41 |
| Female | 1262 | 47.7% | 739 | 46.3% |  |
| **Ethnicity (N=5733)** |  |  |  |  |  |
| Hispanic | 2363 | 73.8% | 2005 | 79.2% | <.001 |
| Non-Hispanic | 837 | 26.2% | 528 | 20.8% |  |
| **Marital Status (N=5812)** |  |  |  |  |  |
| Currently married | 1382 | 42.6% | 1261 | 49.1% | <.001 |
| Other (never married, divorced, widowed) | 1861 | 57.4% | 1308 | 50.9% |  |
| **Awake at night to smoke (N=5665)** |  |  |  |  |  |
| No | 2104 | 66.2% | 1568 | 63.1% | 0.02 |
| Yes | 1076 | 33.8% | 917 | 36.9% |  |
| **Dual Use (N=5610)** |  |  |  |  |  |
| Cig only | 2774 | 88.2% | 2150 | 87.2% | 0.32 |
| Vaping only | 123 | 3.9% | 93 | 3.8% |  |
| Both | 248 | 7.9% | 222 | 9.0% |  |
| **Difficulty sleeping - past (N=5856)** | 120 | 3.70% | 119 | 4.50% | 0.13 |
| **Difficulty sleeping - current (N=5856)** | 663 | 20.50% | 566 | 21.60% | 0.35 |
| **Insomnia as a withdrawal symptom (N=5630)** | 481 | 15.20% | 395 | 16.10% | 0.37 |
|  | **M** | **SD** | **M** | **SD** |  |
| **Age (N=5952)** | 50.94 | 13.73 | 47.79 | 14.63 | <.001 |
| **HSI (N=5541)** | 2.65 | 1.57 | 2.76 | 1.55 | 0.29 |
| *Note:* N=Number, M=Mean, SD=Standard Deviation. | | | | | |

**Appendix Tables 3.** The results of the sensitivity analyses using two additional datasets are presented below. In Table S2-1, missing responses for the pre-existing sleep difficulties variables were coded as 'No sleep difficulties' (0), as missing responses may have resulted from coding errors. In Table S2-2, missing responses for continued tobacco use at the 1- and 7-month follow-ups were coded as 'Yes smoking' (1) under the assumption that participants who did not respond may have continued smoking. The significant results were consistent across the three datasets.

| **Appendix Table 3-1.** **Sensitivity analyses for missing data on sleep difficulties:** **Results from multivariable models examining the associations between pre-existing sleep difficulties and continued tobacco use at 1- and 7-month follow-ups** | | | | | | | | | |
| --- | --- | --- | --- | --- | --- | --- | --- | --- | --- |
|  | **Continued tobacco use at 1-month follow-up** | | | |  | **Continued tobacco use at 7-month follow-up** | | | |
|  |  | **N (%)** | **Unadjusted OR (95% CI)** | **Adjusted OR (95% CI)** |  |  | **N (%)** | **Unadjusted OR (95% CI)** | **Adjusted OR (95% CI)** |
| **Difficulty sleeping - past** | ***No*** | 2000 (71.0%) | ref. | ref. |  | ***No*** | 1053 (74.7%) | ref. | ref. |
|  | ***Yes*** | 77 (79.4%) | 1.57 (0.95, 2.58) | **2.95 (1.28, 6.79)*** |  | ***Yes*** | 46 (92.0%) | **3.89 (1.39, 10.88)*** | **4.64 (1.06, 20.23)*** |
| **Difficulty sleeping - present** | ***No*** | 1680 (71.5%) | ref. | ref. |  | ***No*** | 860 (74.8%) | ref. | ref. |
|  | ***Yes*** | 397 (70.5%) | 0.95 (0.78, 1.17) | 0.88 (0.62, 1.24) |  | ***Yes*** | 239 (77.3%) | 1.15 (0.86, 1.55) | 0.85 (0.54, 1.34) |
| **Difficulty sleeping (past or present)** | ***No*** | 1610 (71.2%) | ref. | ref. |  | ***No*** | 816 (73.9%) | ref. | ref. |
|  | ***Yes*** | 467 (71.6%) | 1.02 (0.84, 1.24) | 1.11 (0.80, 1.55) |  | ***Yes*** | 283 (79.7%) | **1.39 (1.04, 1.86)*** | 1.09 (0.70, 1.71) |
| **Insomnia as a withdrawal symptom** | ***No*** | 1792 (71.9%) | ref. | ref. |  | ***No*** | 951 (75.8%) | ref. | ref. |
|  | ***Yes*** | 285 (67.7%) | 0.82 (0.66, 1.02) | 0.80 (0.54, 1.16) |  | ***Yes*** | 148 (72.5%) | 0.85 (0.61, 1.18) | 0.84 (0.50, 1.43) |
| **Any sleep issue (difficulty past or present, insomnia as withdrawal symptom)** | ***No*** | 1443 (71.4%) | ref. | ref. |  | ***No*** | 734 (74.4%) | ref. | ref. |
|  | ***Yes*** | 634 (71.2%) | 0.99 (0.83, 1.18) | 1.13 (0.83, 1.55) |  | ***Yes*** | 365 (77.2%) | 1.16 (0.90, 1.50) | 0.94 (0.62, 1.44) |
| *Note:* Models were adjusted for sex, age, education, ethnicity, baseline heaviness of smoking index, depression, anxiety, excessive alcohol use, and excessive caffeine use. Continued smoking refers to non-abstinence at each follow-up. | | | | | | | | | |
| Missing responses for the pre-existing sleep difficulties variables were coded as 'No sleep difficulties' (0), as missing responses may have resulted from coding errors. | | | | | | | | | |
| * <.05 ** <.01 ***<.001 | | | | | | | | | |
| **Appendix Table 3-2.** **Sensitivity analyses for missing data on continued tobacco use:** **Results from multivariable models examining the associations between pre-existing sleep difficulties and continued tobacco use at 1- and 7-month follow-ups** | | | | | | | | | |
|  | **Continued tobacco use at 1-month follow-up** | | | |  | **Continued tobacco use at 7-month follow-up** | | | |
|  |  | **N (%)** | **Unadjusted OR (95% CI)** | **Adjusted OR (95% CI)** |  |  | **N (%)** | **Unadjusted OR (95% CI)** | **Adjusted OR (95% CI)** |
| **Difficulty sleeping - past** | ***No*** | 2313 (74.4%) | ref. | ref. |  | ***No*** | 2775 (89.2%) | ref. | ref. |
|  | ***Yes*** | 100 (83.3%) | **1.72 (1.06, 2.80)*** | **2.76 (1.22, 6.24)*** |  | ***Yes*** | 116 (96.7%) | **3.50 (1.28, 9.55)*** | **4.85 (1.15, 20.38)*** |
| **Difficulty sleeping - present** | ***No*** | 1916 (74.6%) | ref. | ref. |  | ***No*** | 2298 (89.5%) | ref. | ref. |
|  | ***Yes*** | 497 (75%) | 1.02 (0.84, 1.24) | 0.87 (0.63, 1.19) |  | ***Yes*** | 593 (89.4%) | 0.99 (0.75, 1.31) | 0.83 (0.54, 1.26) |
| **Difficulty sleeping (past or present)** | ***No*** | 1825 (74.3%) | ref. | ref. |  | ***No*** | 2190 (89.1%) | ref. | ref. |
|  | ***Yes*** | 588 (76.1%) | 1.10 (0.91, 1.33) | 1.07 (0.78, 1.47) |  | ***Yes*** | 701 (90.7%) | 1.19 (0.90, 1.56) | 1.08 (0.71, 1.65) |
| **Insomnia as a withdrawal symptom** | ***No*** | 2023 (75.2%) | ref. | ref. |  | ***No*** | 2407 (89.5%) | ref. | ref. |
|  | ***Yes*** | 345 (71.7%) | 0.84 (0.67, 1.04) | 0.76 (0.53, 1.09) |  | ***Yes*** | 425 (88.4%) | 0.89 (0.66, 1.21) | 0.94 (0.58, 1.55) |
| **Any sleep issue (difficulty past or present, insomnia as withdrawal symptom)** | ***No*** | 1563 (74.5%) | ref. | ref. |  | ***No*** | 1882 (89.7%) | ref. | ref. |
|  | ***Yes*** | 758 (75.1%) | 1.03 (0.88, 1.23) | 1.04 (0.77, 1.41) |  | ***Yes*** | 906 (89.8%) | 1.10 (0.78, 1.29) | 0.95 (0.63, 1.43) |
| *Note:* Models were adjusted for sex, age, education, ethnicity, baseline heaviness of smoking index, depression, anxiety, excessive alcohol use, and excessive caffeine use. Continued smoking refers to non-abstinence at each follow-up. | | | | | | | | | |
| Missing responses for continued tobacco use at the 1- and 7-month follow-ups were coded as 'Yes smoking' (1) under the assumption that participants who did not respond may have continued smoking | | | | | | | | | |
| * <.05 ** <.01 ***<.001 | | | | | | | | | |
